# Supplementary material for: Epigenetic suppression of PGC1α (PPARGC1A) causes collateral sensitivity to HMGCR-inhibitors within BRAF-treatment resistant melanomas
Source: Nat Commun. 2023 Jun 5;14:3251. doi: 10.1038/s41467-023-38968-7 (PMC10241879; doi:10.1038/s41467-023-38968-7)
Supplement: Supplementary file 1 — Supplementary Information [file 41467_2023_38968_MOESM1_ESM.pdf]

Supplementary Fig. 1

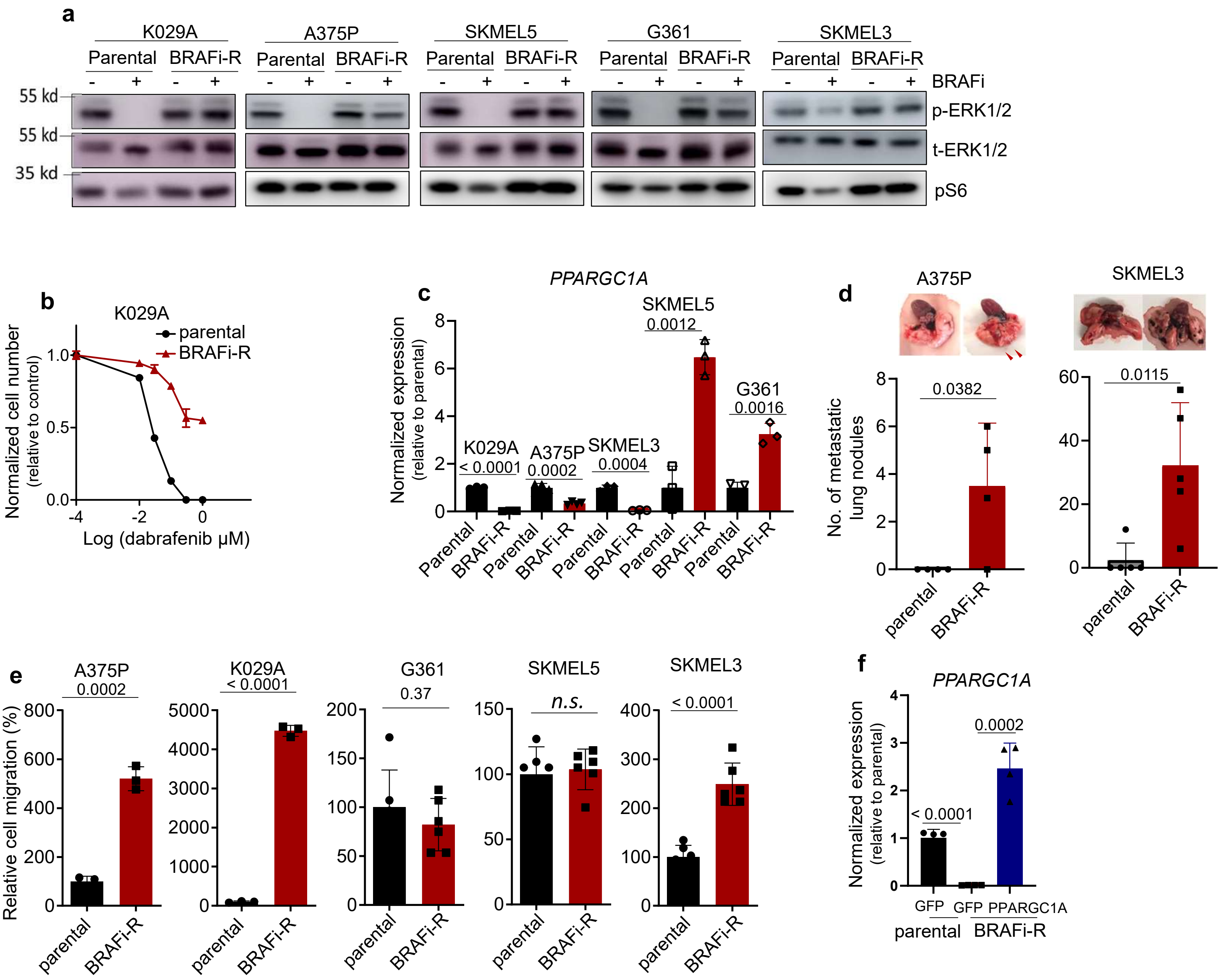

Supplementary Fig. 1: Downregulation of PGC1α in chronic BRAF-inhibitor treated melanoma cells confers an aggressive tumor phenotype.

a. Western blot assays of p-ERK1/2, total-ERK1/2 and pS6 in the parental and chronic BRAF-inhibitor adapted K029A, A375P, SKMEL5, G361, and SKMEL3 cells.

b. Fold-change in cell numbers of parental and chronic BRAF-inhibitor treated K029A cells following treatment with dabrafenib, a BRAF inhibitor, at the indicated concentrations for 1 week (mean ± SEM, n = 3).

c. PPARGC1A expression in parental and chronic BRAF-inhibitor treated K029A, A375P, SKMEL5, G361 and SKMEL3 cells (Significance were calculated with unpaired two-sided t test) (mean ± SEM, n = 3 for K029A, A375P SKMEL5 and G361 cells, and n = 4 for SKMEL3 cells).

d. Representative images and quantification of lung metastases (Significance were calculated with unpaired two-sided t test) (mean ± SEM, n = 4 for A375P model and n = 5 for SKMEL3 model).

e. Trans-well migration assay of parental and chronic treated BRAF-inhibitor adapted K029A, A375P, SKMEL5, G361 and SKMEL3 cells (Significance were calculated with unpaired two-sided t test) (mean ± SEM, n = 3).

f. Fold-change in PPARGC1A mRNA expression levels in parental and chronic treated BRAF-inhibitor adapted K029A cells with GFP or PPARGC1A overexpression (Significance were calculated with unpaired two-sided t test) (mean ± SEM, n = 4).

Supplementary Fig. 2

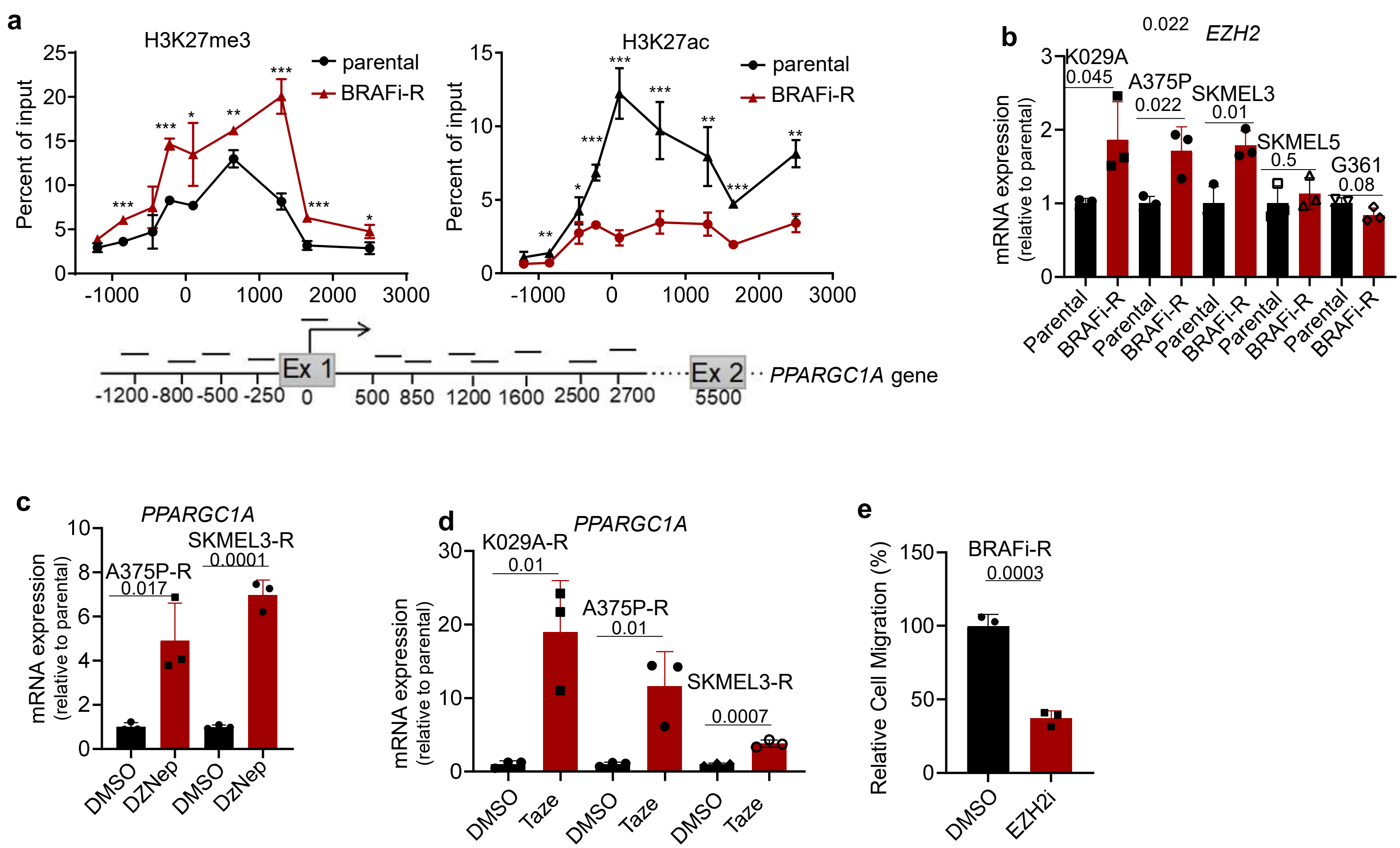

Supplementary Fig. 2: Chronic BRAF-inhibitor treated cells display epigenetic silencing of PPARGC1A expression.

a. ChIP analysis of H3K27me3 and H3K27ac marks across the PPARGC1A promoter region in parental and chronic treated BRAF-inhibitor resistant A375P cells (Significance were calculated with unpaired two-sided t test, p value from left to right for H3K27me3 are 0.057, 0.00007, 0.19, 0.0001, 0.048, 0.006, 0.0007, 0.0008, 0.03 while p value for H3K27ac are 0.1, 0.008, 0.9, 0.0005, 0.0007, 0.007, 0.02, 0.00002, 0.002) (mean ± SEM, n = 3) (* means $p < 0.05$, ** means $p < 0.001$, *** means $P < 0.0001$).

b. Fold-change in EZH2 mRNA expression levels in the parental and chronic treated BRAF-inhibitor adapted K029A, A375P, SKMEL5, G361 and SKMEL3 cells (Significance were calculated with unpaired two-sided t test) (mean ± SEM, n = 3).

c. Fold-change in PPARGC1A mRNA expression levels in the chronic treated BRAF-inhibitor adapted A375P and SKMEL3 cells (1μM, DZNep for 5 days) (Significance were calculated with unpaired two-sided t test) (mean ± SEM, n = 3).

d. Fold-change in PPARGC1A mRNA expression levels in the chronic treated BRAF-inhibitor adapted K029A, A375P and SKMEL3 cells (0.5 μM, tazemetostat for 5 days) (Significance were calculated with unpaired two-sided t test) (mean ± SEM, n = 3).

e. Quantification of trans-well migration assay of chronic BRAF-inhibitor adapted K029A cells treated with EZH2 inhibitor (3μM GSK126) (Significance were calculated with unpaired two-sided t test) (mean ± SEM, n = 3).

Supplementary Fig. 3

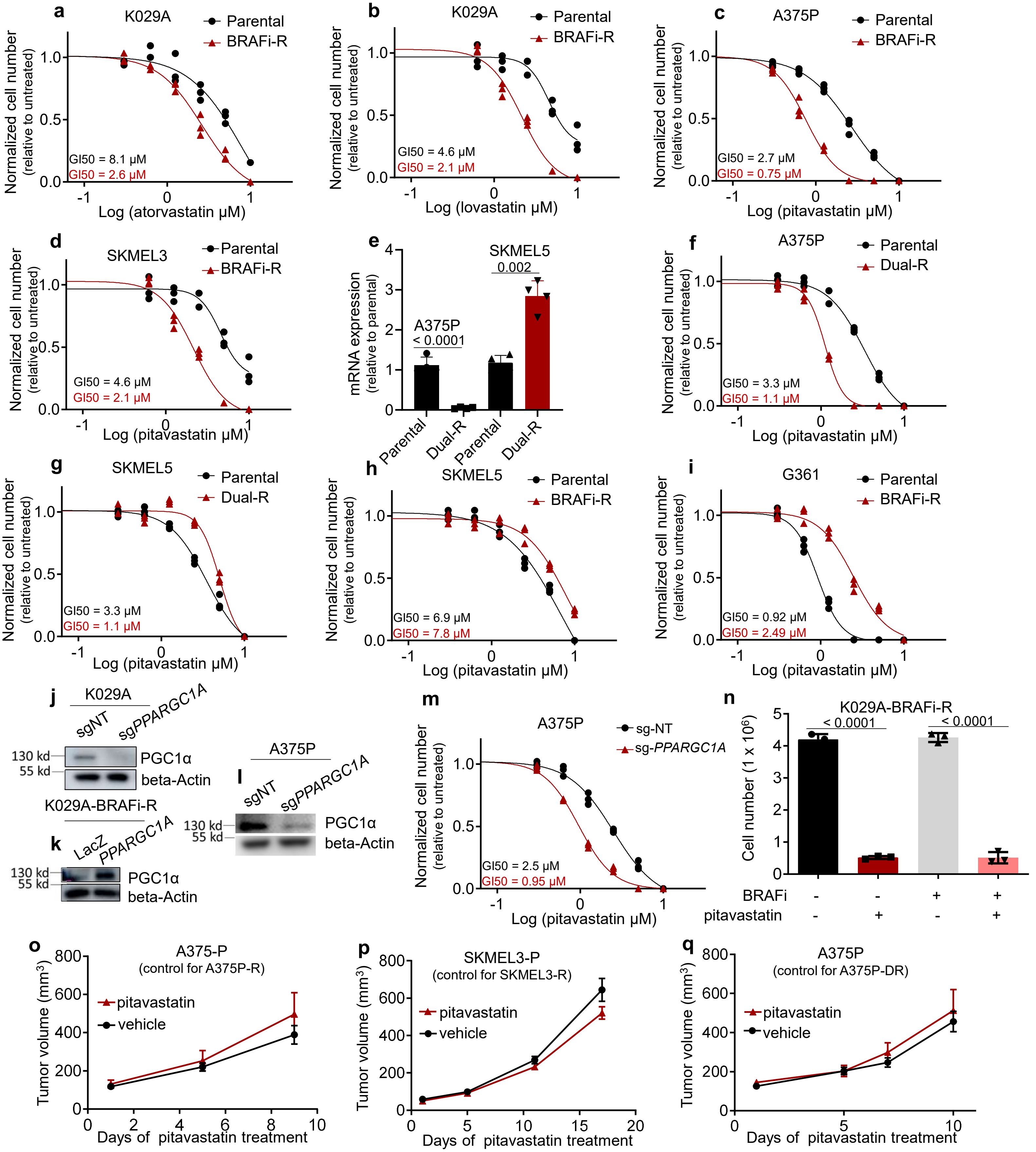

Supplementary Fig. 3: Sensitivity to pitavastatin is dependent on PGC1α level in chronic BRAF-inhibitor treated melanoma cells.

a. Change in cell numbers for parental and chronic BRAF-inhibitor adapted K029A cells treated with atorvastatin at the indicated concentration for 3 days (n = 3).

b. Change in cell numbers for parental and chronic BRAF-inhibitor adapted cells treated with lovastatin at the indicated concentration for 3 days (n = 3).

c. Change in cell numbers for parental and chronic BRAF-inhibitor adapted cells treated with pitavastatin at the indicated concentration for 5 days (n = 3).

d. Change in cell numbers for parental and chronic BRAF-inhibitor adapted SKMEL3 cells treated with pitavastatin at the indicated concentration for 5 days (n = 3).

e. PPARGC1A expression in parental and combinatorial BRAF- and MEK-inhibitor adapted A375P and SKMEL5 cells (mean ± SEM, n = 3). Significance were calculated with unpaired two-sided t test

f. Change in cell numbers for parental and chronic combinatorial BRAF- and MEK-inhibitor adapted A375P cells treated with pitavastatin at the indicated concentration for 5 days (n = 3).

g. Change in cell numbers for parental and chronic combinatorial BRAF- and MEK-inhibitor adapted SKMEL5 cells treated with pitavastatin at the indicated concentration for 5 days (n = 3).

h. Change in cell numbers for parental and chronic BRAF-inhibitor adapted SKMEL5 cells treated with pitavastatin at the indicated concentration for 5 days (n = 3).

i. Change in cell numbers for parental and chronic BRAF-inhibitor adapted G361 cells treated with pitavastatin at the indicated concentration for 5 days (n = 3).

j. Western blot analysis of PGC1α levels in parental K029A cells with/without sgPPARGC1A.

k. Western blot analysis of PGC1α levels in chronic BRAF-inhibitor treated K029A cells with the overexpression of PPARGC1A or LacZ.

l. Western blot analysis of PGC1α levels in parental A375P cells with/without sgPPARGC1A

m. Change in cell numbers for parental A375P cells with/without sgPPARGC1A treated with pitavastatin at the indicated concentration for 5 days (n = 3).

n. Change in cell numbers for chronic BRAF-inhibitor adapted K029A cells treated with pitavastatin (1μM) and/or PLX4032 (1μM) for 72h (mean ± SEM, n = 3). Significance were calculated with unpaired two-sided t test

o. Treatment of xenograft tumors established from parental A375P cells (s.c. implanted in nu/nu mice; control for chronic-treated BRAF-inhibitor resistant A375P cells) using pitavastatin administration at 1 mg/kg b.i.d., (mean ± SEM, n = 10). Significance calculated as un-paired, two-sided t test.

p. Treatment of xenograft tumors established from SKMEL3 parental cells (s.c. implanted in nu/nu mice; control for chronic-treated BRAF-inhibitor resistant A375P cells) using pitavastatin administration at 1 mg/kg b.i.d., (mean ± SEM, n = 5). Significance calculated as un-paired, two-sided t test.

q. Treatment of xenografted tumors established from A375P parental cells (s.c. implanted in nu/nu mice; control for chronic-treated BRAF-inhibitor and MEK-inhibitor resistant A375P cells) using pitavastatin administration at 1 mg/kg b.i.d., (mean ± SEM, n = 10). Significance calculated as un-paired, two-sided t test.

Supplementary Fig. 4

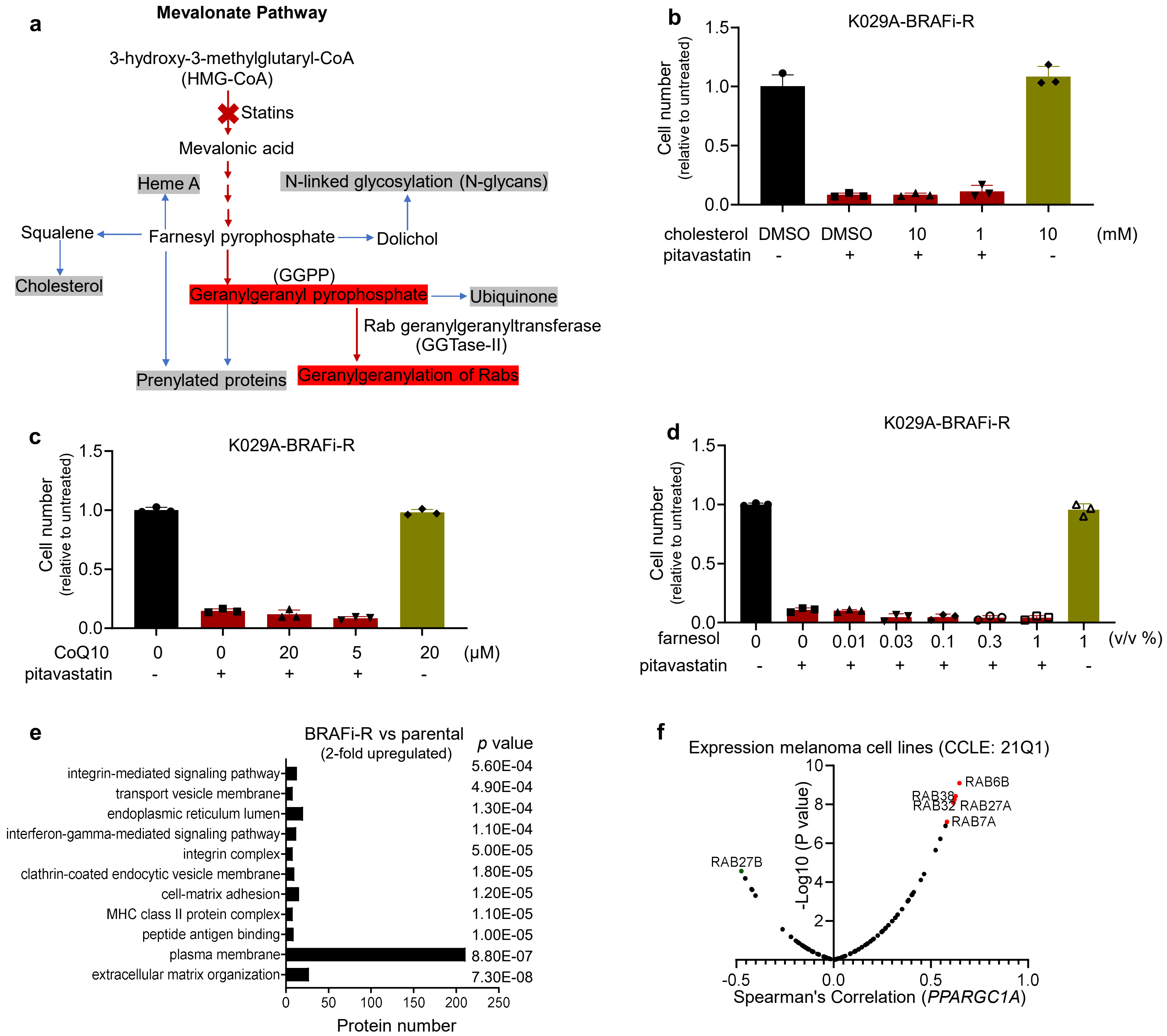

Supplementary Fig. 4: The sensitivity of chronic treated BRAF-inhibitor adapted melanoma cells to pitavastatin is mediated through GGPP mediated prenylation of proteins.

a. Schematic diagram of mevalonate pathway. Highlighted in red are the biosynthetic components downstream of mevalonate (mevalonic acid) that are able to rescue the cell growth effects of HMGCR-inhibition.

b. Change in cell numbers for chronic BRAF-inhibitor adapted K029A cells treated with 1μM pitavastatin and cholesterol at the indicated concentrations for 72 h (mean ± SEM, n = 3).

c. Change in cell numbers for chronic BRAF-inhibitor adapted K029A cells treated with 1μM pitavastatin and CoQ10 at the indicated concentrations for 72 h (mean ± SEM, n = 3).

d. Change in cell numbers for chronic BRAF-inhibitor adapted K029A cells treated with 1μM pitavastatin and farnesol at the indicated concentrations for 72h (mean ± SEM, n = 3).

e. Histogram of protein gene ontology (GO) enrichment analysis (observed change > 2-fold, and associated p values (Significance calculated with Fisher's Exact test adopted by DAVID Bioinformatics Resources) within parental and chronic BRAF-inhibitor treated K029A cells.

f. Correlation between PPARGC1A and RAB family members by expression levels across melanoma cell lines (Significance calculated as unpaired two-sided spearman's correlation analysis) (CCLE - Public 21Q1).

Supplementary Fig. 5

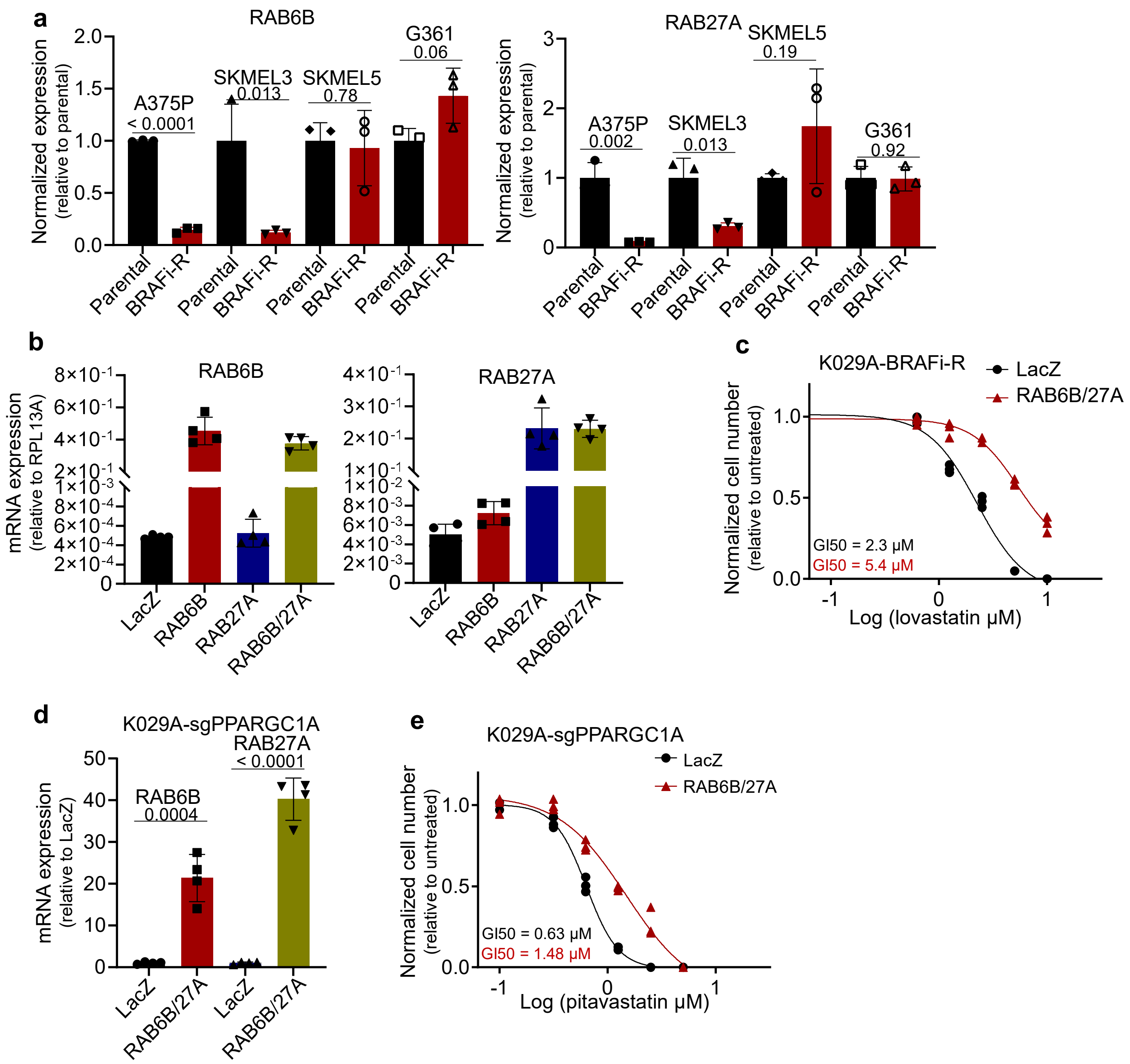

Supplementary Fig. 5: Chronic BRAF-inhibitor treated melanoma cells depend on RAB6B and RAB27A for pitavastatin sensitivity.

a. Expression levels of RAB6B and RAB27A in parental and chronic BRAF-inhibitor treated A375P, SKMEL5, G361 and SKMEL3 cells (Significance were calculated with unpaired two-sided t test between the paired groups) (mean ± SEM, n = 3).

b. Expression of RAB6B and RAB27A in chronic BRAF-inhibitor treated K029A cells with overexpression of LacZ, RAB6B, RAB27A or combinatorial RAB6B and RAB27A (mean ± SEM, n = 3).

c. Change in cell numbers for chronic BRAF-inhibitor adapted K029A cells with ectopic expression of LacZ or dual RAB6B and RAB27A following treatment with lovastatin at the indicated concentration for 72h. Fold change in cell numbers were normalized by comparing cell numbers to mock (DMSO) treated (n =3).

d. Expression levels of RAB6B and RAB27A within K029A-sgPPARGC1A cells with overexpression of LacZ or combinatorial RAB6B and RAB27A (Significance were calculated with unpaired two-sided t test between the paired groups) (mean ± SEM, n = 3).

e. Change in cell numbers for K029A-sgPPARGC1A cells with ectopic expression of LacZ or combinatorial RAB6B and RAB27A following treatment with pitavastatin at the indicated concentration for 72h. Fold changes were normalized by comparing cell numbers to mock (DMSO) treated (n =3).

Supplementary Fig. 6

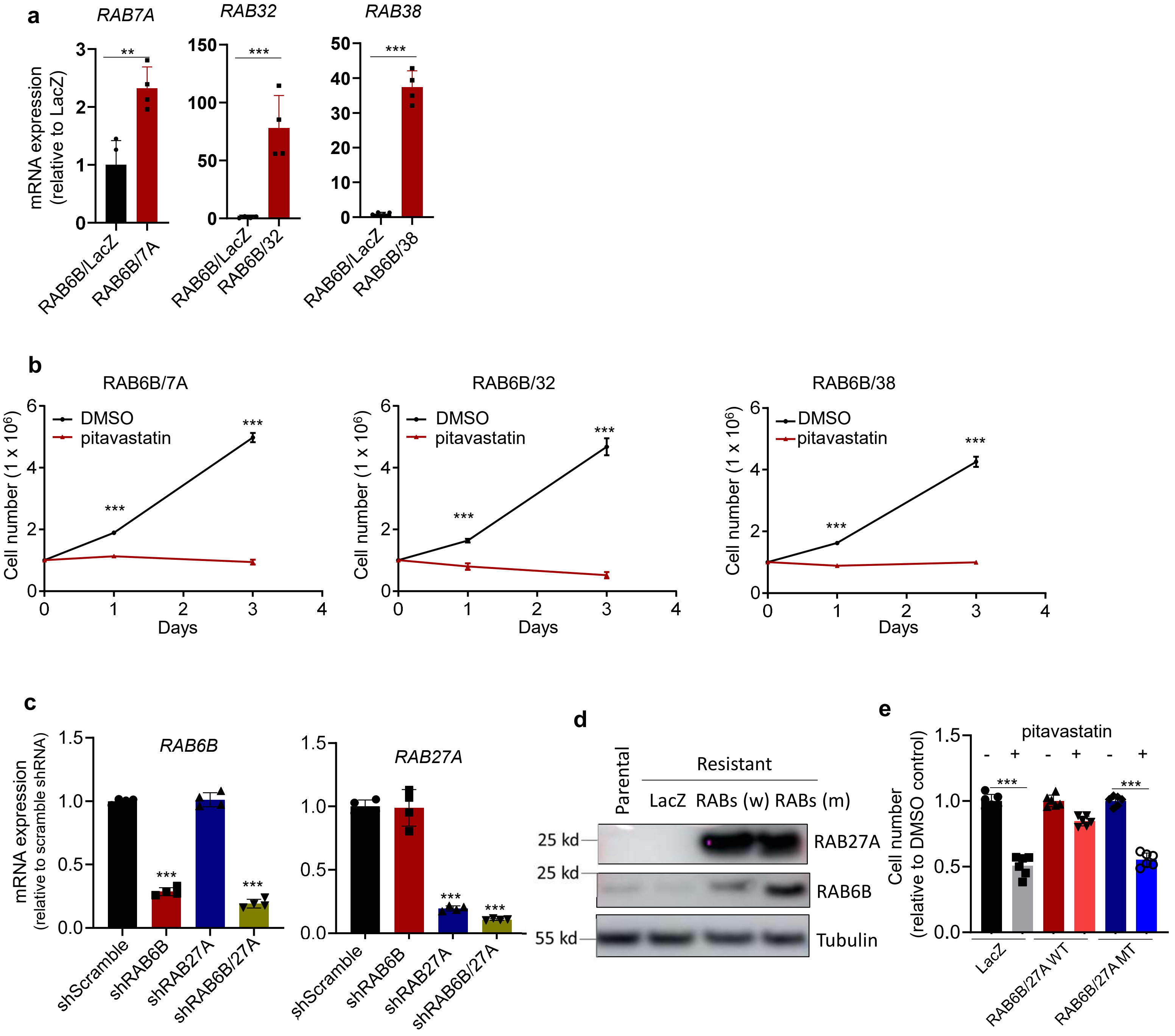

Supplementary Fig. 6: Ectopic RAB6B and RAB27A expression, but not RAB6B with RAB7A, RAB32, or RAB38, nor RAB6B and RAB27A prenylation site-mutants, rescues chronic BRAF-inhibitor adapted cells from pitavastatin inhibition of cell growth.

a. Expression levels of RAB7A, RAB32 and RAB38 in chronic BRAF-inhibitor adapted K029A cells with ectopic expression RAB7A/RAB6B, RAB32/RAB6B and RAB38/RAB6B respectively (mean ± SEM, n = 3). Significance calculated with unpaired two-sided t test.

b. Effect on chronic BRAF-inhibitor adapted K029A cells with ectopic expression RAB6B+RAB7A, RAB6B+RAB32, and RAB6B+RAB38 cell growth following treatment with 1μM pitavastatin (mean ± SEM, n = 3).

c. Expression levels of RAB6B and RAB27A in parental K029A cells with the shRNA-mediated knockdown of RAB6B, RAB27A or combinatorial RAB6B and RAB27A (mean ± SEM, n = 4). Significance were calculated with unpaired two-sided t test

d. Western blot analyses detail expression of RAB6B and RAB27A in parental and chronic BRAF-inhibitor adapted A375P cells with ectopic wild-type or prenylation site-mutant alleles of RAB6B and RAB27A.

e. Change in cell numbers for parental A375P cells with ectopic expression of LacZ (control), prenylation mutant RAB6B and RAB27A, or wild-type RAB6B and RAB27A treated with 0.5μM pitavastatin for 72 h (mean ± SEM, n = 3). Significance calculated with unpaired two-sided t test.

Supplementary Fig. 7

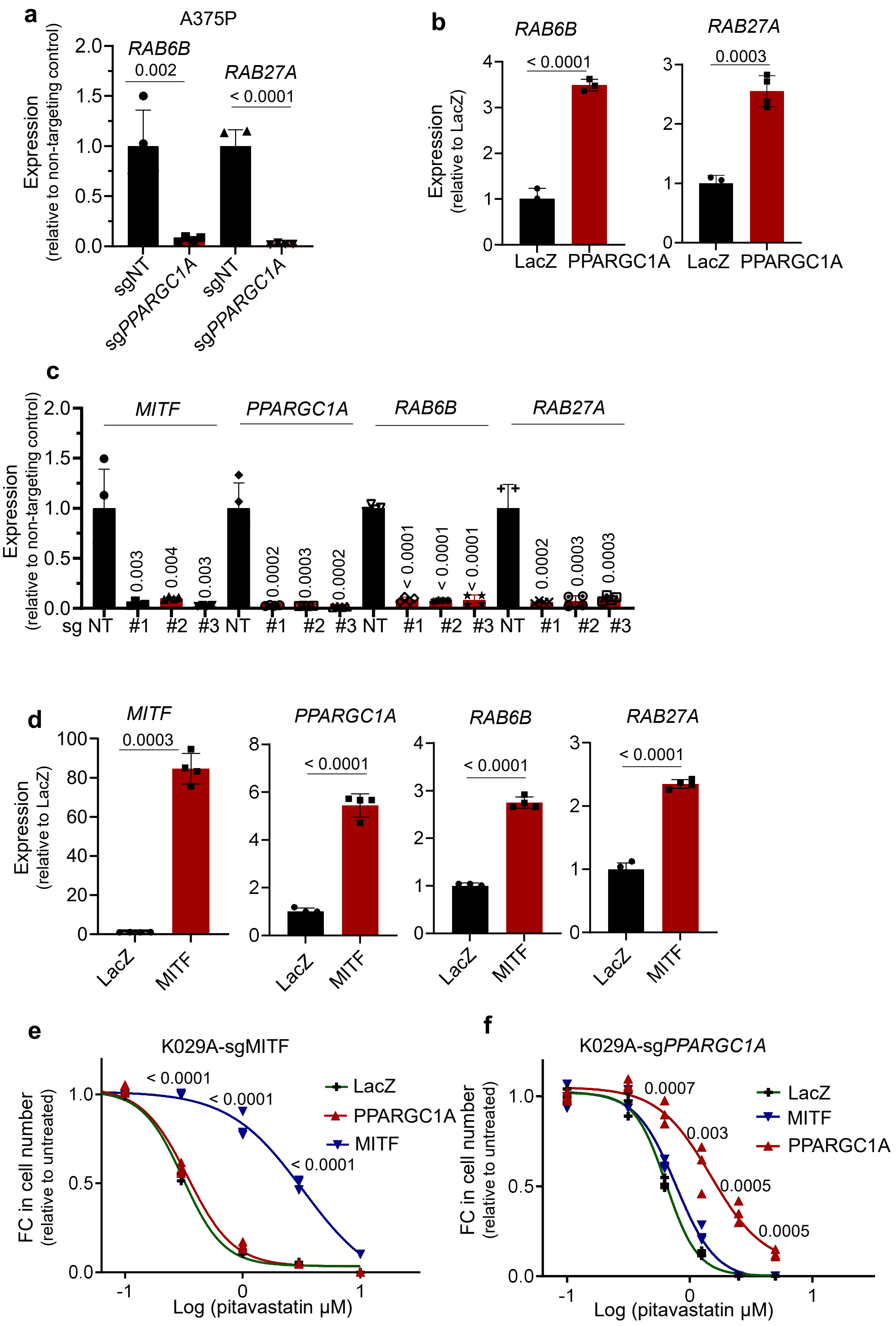

Supplementary Fig. 7: RAB6B and RAB27A expression levels are regulated by PPARGC1A and MITF.

a. Expression levels of RAB6B and RAB27A in parental A375P cells with/without sgPPARGC1A (mean ± SEM, n = 4). Significance calculated with unpaired two-sided t test.

b. Expression levels of RAB6B and RAB27A in chronic BRAF-inhibitor adapted K029A cells with ectopic LacZ or PPARGC1A (mean ± SEM, n = 4). Significance calculated with unpaired two-sided t test.

c. Expression levels of MITF, PPARGC1A, RAB27A, RAB6B in parental K029A cells with/without sgMITF (three different sgRNA were used) (mean ± SEM, n = 4). Significance calculated with unpaired two-sided t test.

d. Expression levels of MITF, PPARGC1A, RAB27A, RAB6B in parental K029A cells with sgMITF and reconstituted ectopic LacZ or MITF (mean ± SEM, n = 4). Significance calculated with unpaired two-sided t test.

e. Change in cell numbers for parental K029A cells with sgMITF and reconstituted LacZ, MITF or PPARGC1A treated with pitavastatin at the indicated concentration for 72 h (n = 4). Significance calculated with unpaired two-sided t test.

f. Change in cell numbers for parental K029A cells with sgPPARGC1A and reconstitution with ectopic LacZ, MITF or PPARGC1A. Cells were treated with pitavastatin at the indicated concentration for 72 h (n = 4). Significance calculated with unpaired two-sided t test.

Supplementary Fig. 8

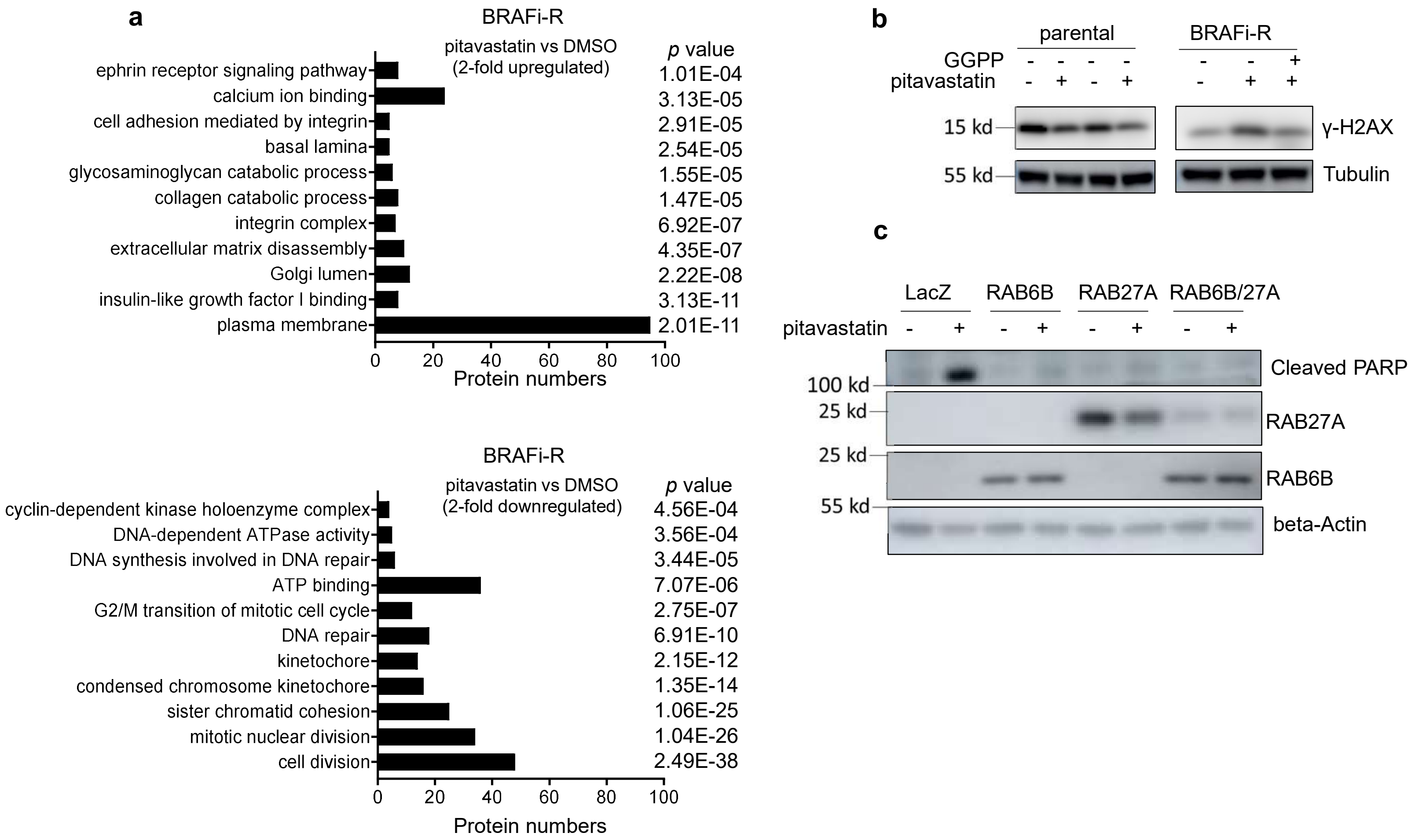

Supplementary Fig. 8: HMGCR inhibitor treatment induces G0/G1 arrest and markers of DNA damage.

a. Gene Ontology (GO) enrichment analysis of the significantly changed proteins (pitavastatin vs DMSO) in K029A chronic treated BRAF-inhibitor adapted cells (p values were calculated with Fisher's Exact test adopted by DAVID Bioinformatics Resources).

b. Western blot analyses of gamma-H2AX in parental and chronic BRAF-inhibitor adapted K029A cells treated with 1μM pitavastatin for 24h.

c. Western blot analyses of cleaved PARP, RAB6B and RAB27A levels in chronic BRAF-inhibitor adapted K029A cells with ectopic expression of LacZ or combinatorial RAB27A and RAB6B treated with 1μM pitavastatin for 24h.

Supplementary Fig. 9

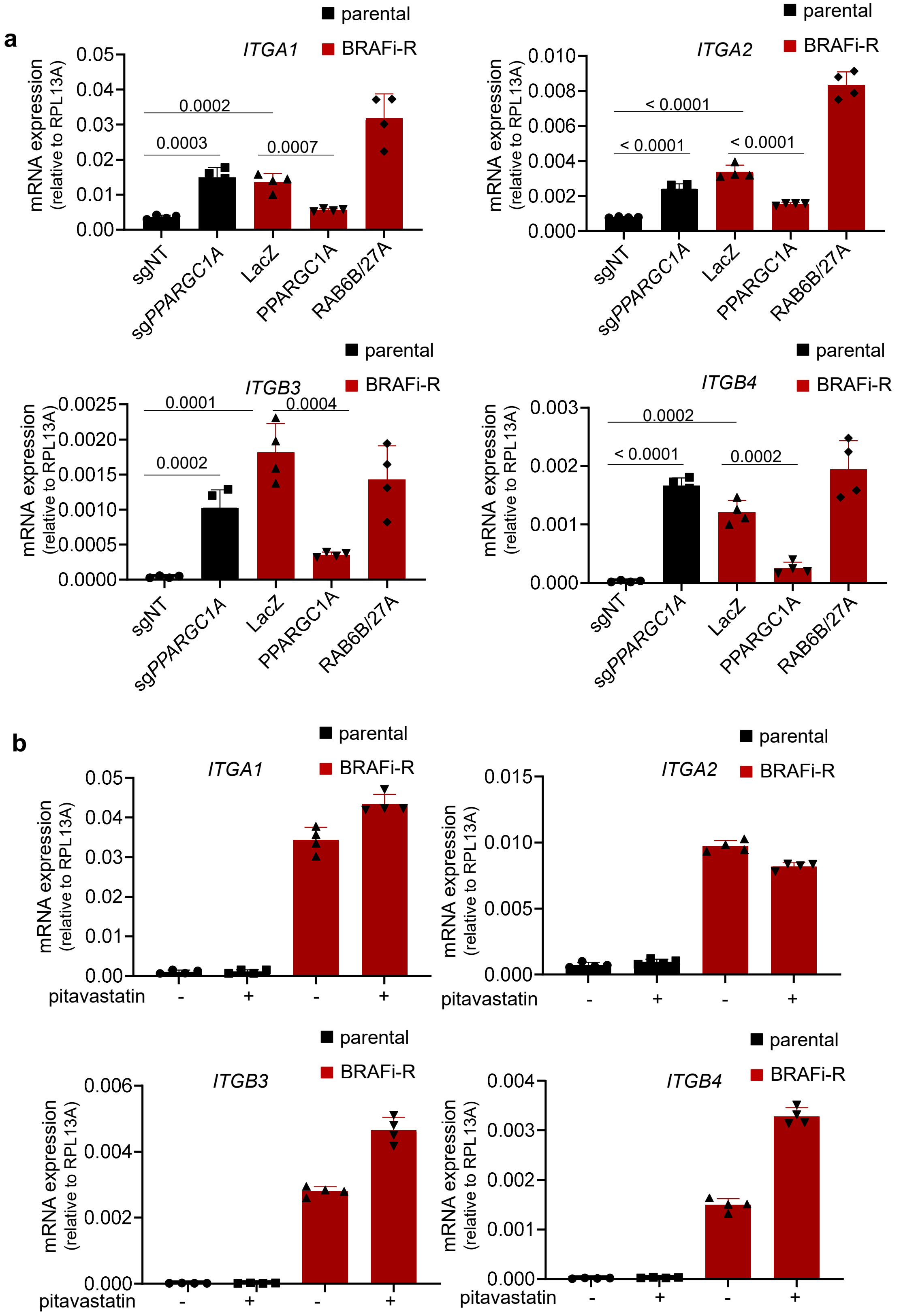

Supplementary Fig. 9: Expression of integrins in K029A parental and chronic treated BRAF-inhibitor adapted cells.

a. Expression levels of integrins across parental (without or with sgPPARGC1A) and chronic BRAF-inhibitor adapted K029A cells (with ectopic LacZ, PPARGC1A, or combinatorial RAB6B and RAB27A) (mean ± SEM, n = 3).

b. Expression levels of integrins across parental and chronic BRAF-inhibitor adapted K029A cells following treatment with 1μM pitavastatin for 24h (mean ± SEM, n = 3).

Supplementary Fig. 10

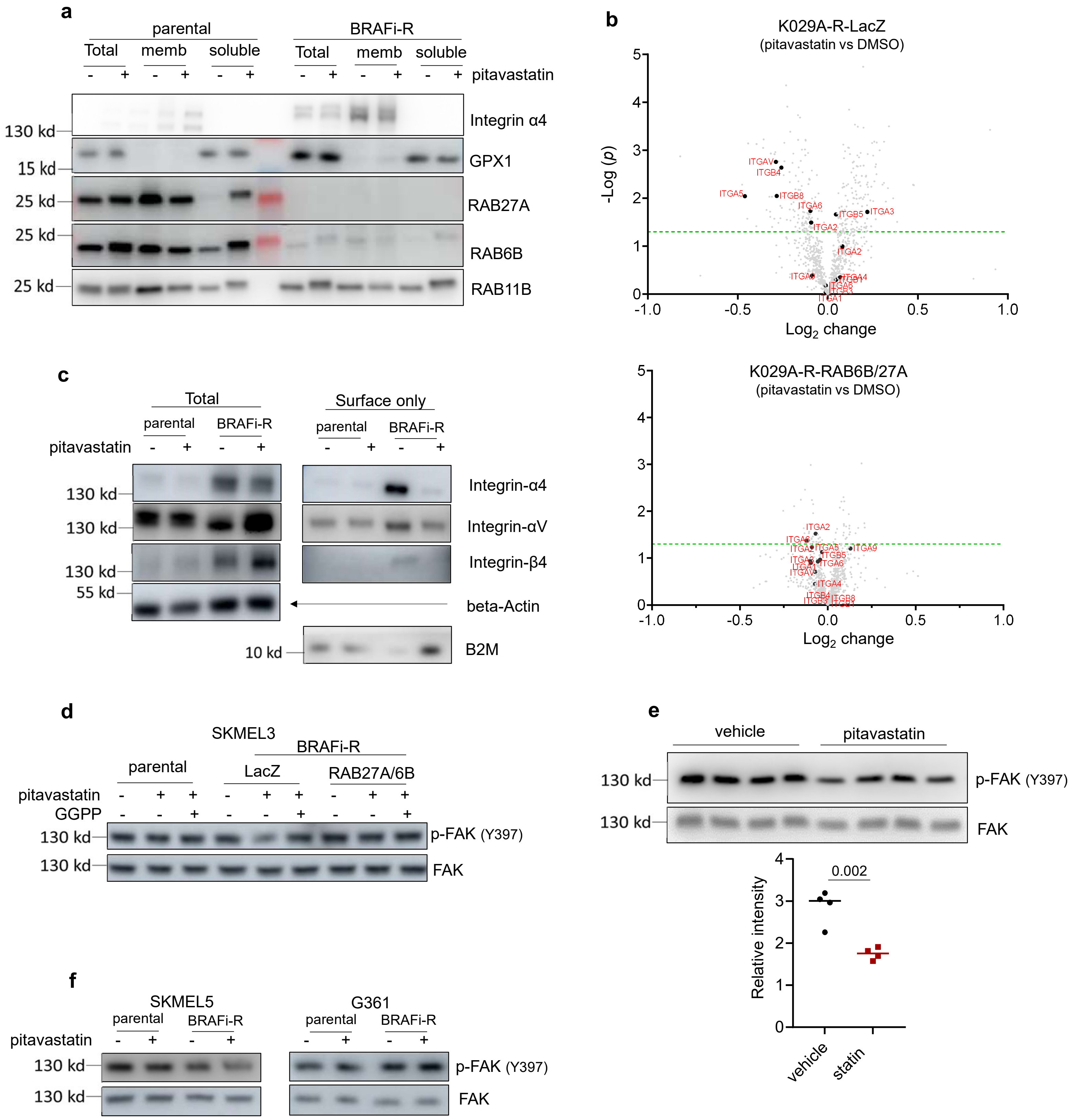

Supplementary Fig. 10: HMGCR inhibitor treatment reduces RAB6B and RAB27A membrane association and integrin receptor cell surface retention.

a. Western blot analyses of integrin-α4 (a positive control for membrane attached/located protein), GPX1 (a negative control for membrane attached/located protein), RAB27A, RAB6B and RAB11B in parental and chronic BRAF-inhibitor adapted K029A cells treated with 1μM pitavastatin for 24h. The membrane fraction and soluble fraction were separated by ultra-centrifugation.

b. Volcano plot of plasma membrane mass spectrometric proteomic data from chronic BRAF-inhibitor adapted cells K029A following treatment with pitavastatin for 24h. *p* value (two sides t test) plotted in a log10 scale and protein fold changes (pitavastatin vs DMSO) plotted in a log2 scale. Significance calculated with unpaired two-sided t test.

c. Western blot analyses of plasma membrane associated integrin-α4, integrin-αV and integrin-β4 in the parental and chronic BRAF-inhibitor adapted K029A cells. Cells were treated with 1μM pitavastatin for 24h.

d. Western blot analyses of FAK-pY397 and total FAK across parental and chronic BRAF-inhibitor adapted SKMEL3 cells with ectopic LacZ or combinatorial RAB6B and RAB27A. Cells were treated with/without pitavastatin for 24h in combination with/without GGPP.

e. Western blot analyses of FAK-pY397 and total FAK in xenografted tumors from chronic BRAF-inhibitor adapted K029A cells treated with/without pitavastatin at 1mg/kg b.i.d. (n = 4). Significance calculated with unpaired two-sided t test.

Supplementary Fig. 11

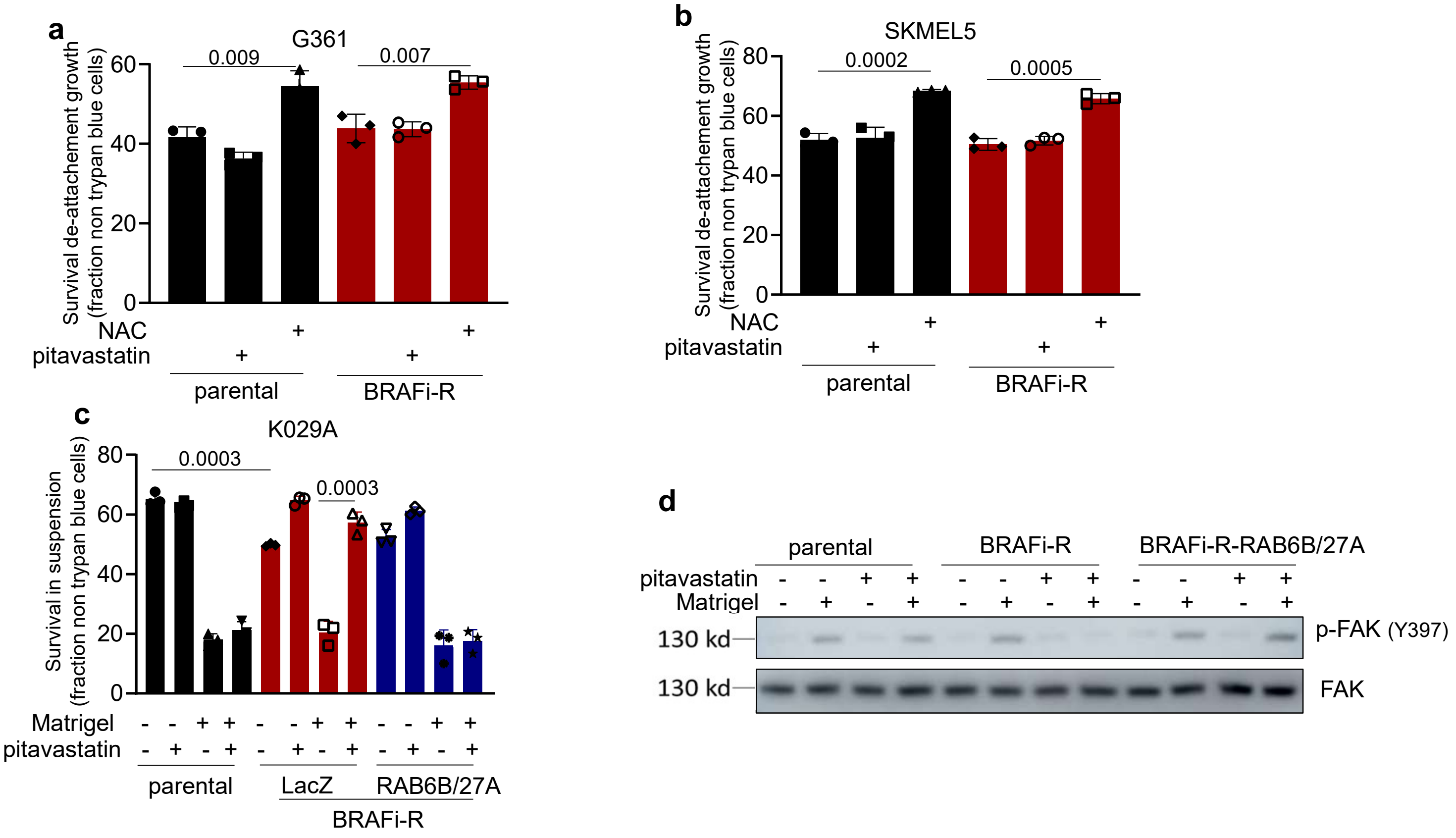

Supplementary Fig. 11: Sensitivity to anoikis and to HMGCR inhibitor are functionally seperable.

a & b. Percentage live cells across parental and chronic BRAF-inhibitor adapted (i) G361 and (j) SKMEL5 cells following suspension in methylcellulose and treatment with/without NAC or pitavastatin (mean ± SEM, n = 3). Significance calculated between the compared groups with unpaired two-sided t test.

c. Percentage live parental and chronic BRAF-inhibitor adapted K029A cells with ectopic LacZ or combinatorial RAB6B and RAB27A following suspension in methylcellulose with/without Matrigel matrix and treatment with/without pitavastatin (mean ± SEM, n = 3). Significance calculated between the compared groups with unpaired two-sided t test.

d. Western blot analyses of FAK-pY397 and total FAK in parental and chronic treated BRAF-inhibitor adapted K029A cells with ectopic LacZ or combinatorial RAB6B and RAB27A suspended in methylcellulose with/without Matrigel matrix and treated with/without pitavastatin.
