# Supplementary material for: Epigenetic suppression of PGC1α (PPARGC1A) causes collateral sensitivity to HMGCR-inhibitors within BRAF-treatment resistant melanomas
Source: Nat Commun. 2023 Jun 5;14:3251. doi: 10.1038/s41467-023-38968-7 (PMC10241879; doi:10.1038/s41467-023-38968-7)
Supplement: Supplementary file 3 — Description of Additional Supplementary Files [file 41467_2023_38968_MOESM3_ESM.pdf]

Description of Additional Supplementary Files

File Name: Supplementary Data 1
Description: The names of compounds used for metabolism-focused small compound screening.

File Name: Supplementary Data 2
Description: The primers used for qPCR and amplification of gene fragments for Gateway system, and the sequence of single guide RNA for CRISPR-Cas9 mediated gene knockout.
