# Supplementary material for: Epigenetic suppression of PGC1α (PPARGC1A) causes collateral sensitivity to HMGCR-inhibitors within BRAF-treatment resistant melanomas
Source: Nat Commun. 2023 Jun 5;14:3251. doi: 10.1038/s41467-023-38968-7 (PMC10241879; doi:10.1038/s41467-023-38968-7)
Supplement: Supplementary file 6 — Reporting Summary [file 41467_2023_38968_MOESM6_ESM.pdf]

nature portfolio

Corresponding author(s): Pere Puigserver
Hans Widlund

Last updated by author(s): Apr 30, 2023

Reporting Summary

Nature Portfolio wishes to improve the reproducibility of the work that we publish. This form provides structure for consistency and transparency in reporting. For further information on Nature Portfolio policies, see our Editorial Policies and the Editorial Policy Checklist.

Statistics

For all statistical analyses, confirm that the following items are present in the figure legend, table legend, main text, or Methods section.

n/a	Confirmed	
☐	☒	The exact sample size (*n*) for each experimental group/condition, given as a discrete number and unit of measurement
☐	☒	A statement on whether measurements were taken from distinct samples or whether the same sample was measured repeatedly
☐	☒	The statistical test(s) used AND whether they are one- or two-sided<br>*Only common tests should be described solely by name; describe more complex techniques in the Methods section.*
☐	☒	A description of all covariates tested
☐	☒	A description of any assumptions or corrections, such as tests of normality and adjustment for multiple comparisons
☐	☒	A full description of the statistical parameters including central tendency (e.g. means) or other basic estimates (e.g. regression coefficient) AND variation (e.g. standard deviation) or associated estimates of uncertainty (e.g. confidence intervals)
☐	☒	For null hypothesis testing, the test statistic (e.g. *F*, *t*, *r*) with confidence intervals, effect sizes, degrees of freedom and *P* value noted<br>*Give P values as exact values whenever suitable.*
☒	☐	For Bayesian analysis, information on the choice of priors and Markov chain Monte Carlo settings
☒	☐	For hierarchical and complex designs, identification of the appropriate level for tests and full reporting of outcomes
☐	☒	Estimates of effect sizes (e.g. Cohen's *d*, Pearson's *r*), indicating how they were calculated

Our web collection on statistics for biologists contains articles on many of the points above.

Software and code

Policy information about availability of computer code

Data collection	There is not specific software uesd for data collection.
Data analysis	We used Excel and GraphPad Prism 9.5.1 for data analysis.

For manuscripts utilizing custom algorithms or software that are central to the research but not yet described in published literature, software must be made available to editors and reviewers. We strongly encourage code deposition in a community repository (e.g. GitHub). See the Nature Portfolio guidelines for submitting code & software for further information.

Data

Policy information about availability of data

All manuscripts must include a data availability statement. This statement should provide the following information, where applicable:

- Accession codes, unique identifiers, or web links for publicly available datasets
- A description of any restrictions on data availability
- For clinical datasets or third party data, please ensure that the statement adheres to our policy

All data supporting the finding of this study are available in the paper and its supplementary information files.
The source data are provided as a Source Data file. The authors declare that all other data supporting the findings of this study are available within the paper and its supplementary information files. The data that support the findings of this study are available from the corresponding author upon reasonable request.
Proteomic data are available in Pride, accession PXD041952.

Human research participants

Policy information about studies involving human research participants and Sex and Gender in Research.

Reporting on sex and gender	N/A
Population characteristics	N/A
Recruitment	N/A
Ethics oversight	N/A

Note that full information on the approval of the study protocol must also be provided in the manuscript.

Field-specific reporting

Please select the one below that is the best fit for your research. If you are not sure, read the appropriate sections before making your selection.

☒ Life sciences ☐ Behavioural & social sciences ☐ Ecological, evolutionary & environmental sciences

For a reference copy of the document with all sections, see nature.com/documents/nr-reporting-summary-flat.pdf

Life sciences study design

All studies must disclose on these points even when the disclosure is negative.

Sample size	Sample sizes were not pre-calucalated but are comparable to similar studies in the field. Sample size and number of independent experiments are stated in the figure captions or in the methods section and Source Data file.<br>An n of 5 was used for in vivo studies whereas for in vitro studies a minimum of an n of 3 was used where sample was limiting and if possible an n of 5 or greater was used.
Data exclusions	no data were excluded.
Replication	All attempts at replication were successful. Experiments were repeated at least 3 times for in vitro experiments and 5 times for in vivo studies.
Randomization	For in vitro experiments sample groups were allocated randomly.<br>Animals and in vitro cultures were paired and distributed into control and experimental groups for side-by-side comparison. The animals were allocated to each group randomly for cell injection.
Blinding	The investigators should keep careful track of protocols because most of the experiments needed multiple treatments (including formulation, cells or drug treatments, sample collection, and so on), and the animal care providers need to be aware of drug treatment. Hence, it would be difficult to blind the investigators to group allocation during data collection and analysis.

Reporting for specific materials, systems and methods

We require information from authors about some types of materials, experimental systems and methods used in many studies. Here, indicate whether each material, system or method listed is relevant to your study. If you are not sure if a list item applies to your research, read the appropriate section before selecting a response.

Materials & experimental systems

n/a	Involved in the study	
☐	☒	Antibodies
☐	☒	Eukaryotic cell lines
☒	☐	Palaeontology and archaeology
☐	☒	Animals and other organisms
☒	☐	Clinical data
☒	☐	Dual use research of concern

Methods

n/a	Involved in the study	
☒	☐	ChIP-seq
☒	☐	Flow cytometry
☒	☐	MRI-based neuroimaging

Antibodies

Antibodies used	RAB6B antibody (PA598909) was purchased from Thermo Fisher Scientific. Cyclin D1 antibody 376 (92G2), Rabbit mAb (2978S),

Antibodies used	Rab11b antibody (2414), Phospho-FAK antibody (Tyr397) , Integrin beta-1 antibody (4706), Integrin alpha-V antibody (4711), Integrin beta-3 antibody (4702) were purchased from Cell Signaling Technology. Integrin α1 antibody (SC-271034), Rap1A antibody (SC-373968), PGC1α antibody (SC-518025), RAB27A antibody (SC-74586) were purchased from Santa Cruz Biotechnology. H3 (ab1791) antibody, H3K27me3 antibody (ab192985), H3K27ac antibody (ab177178) were purchased from Abcam.
Validation	The specificity of each antibody was validated by the manufacturer and provided on the website. RAB6B antibody (PA598909) https://www.thermofisher.com/antibody/product/RAB6B-Antibody-Polyclonal/PA5-98909<br>Cyclin D1 antibody 376 (92G2) https://www.cellsignal.com/products/primary-antibodies/cyclin-d1-92g2-rabbit-mab/2978<br>Rab11b antibody (2414),<br>https://www.cellsignal.com/products/primary-antibodies/rab11b-antibody/2414?site-search-type=Products&N=4294956287&Ntt=rab11b+antibody+%282414%29&fromPage=plp&_requestid=567051<br>Phospho-FAK antibody (Tyr397) https://www.cellsignal.com/products/primary-antibodies/phospho-fak-tyr397-d20b1-rabbit-mab/8556?site-search-type=Products&N=4294956287&Ntt=phospho-fak+antibody+%28tyr397%29&fromPage=plp<br>Integrin beta-1 antibody (4706) https://www.cellsignal.com/products/primary-antibodies/integrin-b1-antibody/4706?site-search-type=Products&N=4294956287&Ntt=integrin+beta-1+antibody+%284706&fromPage=plp&_requestid=567138<br>Integrin alpha-V antibody (4711) https://www.cellsignal.com/products/primary-antibodies/integrin-av-antibody/4711?site-search-type=Products&N=4294956287&Ntt=integrin+alpha-v+antibody+%284711%29&fromPage=plp&_requestid=567164<br>Integrin beta-3 antibody (4702) https://www.cellsignal.com/products/primary-antibodies/integrin-b3-antibody/4702?site-search-type=Products&N=4294956287&Ntt=integrin+beta-3+antibody+%284702%29&fromPage=plp&_requestid=567199<br>RAB27A antibody (SC-74586) https://www.scbt.com/p/rab-27a-antibody-e-8<br>Integrin α1 antibody (SC-271034) https://www.scbt.com/p/integrin-alpha1-antibody-a-9?requestFrom=search<br>Rap1A antibody (SC-373968) https://datasheets.scbt.com/sc-373968.pdf<br>PGC1α antibody (SC-518025) https://datasheets.scbt.com/sc-518025.pdf<br>H3 (ab1791) antibody https://www.abcam.com/products/primary-antibodies/histone-h3-antibody-nuclear-marker-and-chip-grade-ab1791.html<br>H3K27me3 antibody (ab192985) https://www.abcam.com/products/primary-antibodies/histone-h3-tri-methyl-k27-antibody-epr18607-chip-grade-ab192985.html<br>H3K27ac antibody (ab177178) https://www.abcam.com/products/primary-antibodies/histone-h3-acetyl-k27-antibody-ep16602-chip-grade-ab177178.html

Eukaryotic cell lines

Policy information about cell lines and Sex and Gender in Research

Cell line source(s)	All human melanoma cell lines were obtained from the Broad Institute CCLE collection.
Authentication	All human melanoma cell lines were authenticated using small tandem repeat profiling.
Mycoplasma contamination	The cells were tested negative.
Commonly misidentified lines (See ICLAC register)	No cell lines used in this work are listed as commonly misidentified by ICLAC.

Animals and other research organisms

Policy information about studies involving animals; ARRIVE guidelines recommended for reporting animal research, and Sex and Gender in Research

Laboratory animals	Mouse
Wild animals	NA
Reporting on sex	Female and male.
Field-collected samples	NA
Ethics oversight	All animal studies and procedures were conducted according to a protocol approved by the Institutional Animal Care and Use Commitee (IACUC) at at BIDMC. Institutional Animal Care & Use Committee RN-150D. (IACUC protocol number: 013-2021; title: Metabolic Regulation and Vulnerability of Malignant Tumors). All the procedures that involve handling and use of mice in the experiments proposed in this grant application will be in strict accordance with the policies and guidelines established by the Beth Israel Deaconess Medical Center Animal Research Facility which is an AAALAC accredited (Association for Assessment and Accreditation of Lab Animal Care) and PHS Assurance with Office of Laboratory Animal Welfare (OLAW) and complies with all Federal, State and Local laws.

Note that full information on the approval of the study protocol must also be provided in the manuscript.
