# Supplementary material for: Epigenetic suppression of PGC1α (PPARGC1A) causes collateral sensitivity to HMGCR-inhibitors within BRAF-treatment resistant melanomas
Source: Nat Commun. 2023 Jun 5;14:3251. doi: 10.1038/s41467-023-38968-7 (PMC10241879; doi:10.1038/s41467-023-38968-7)

Extended Data Fig. 1

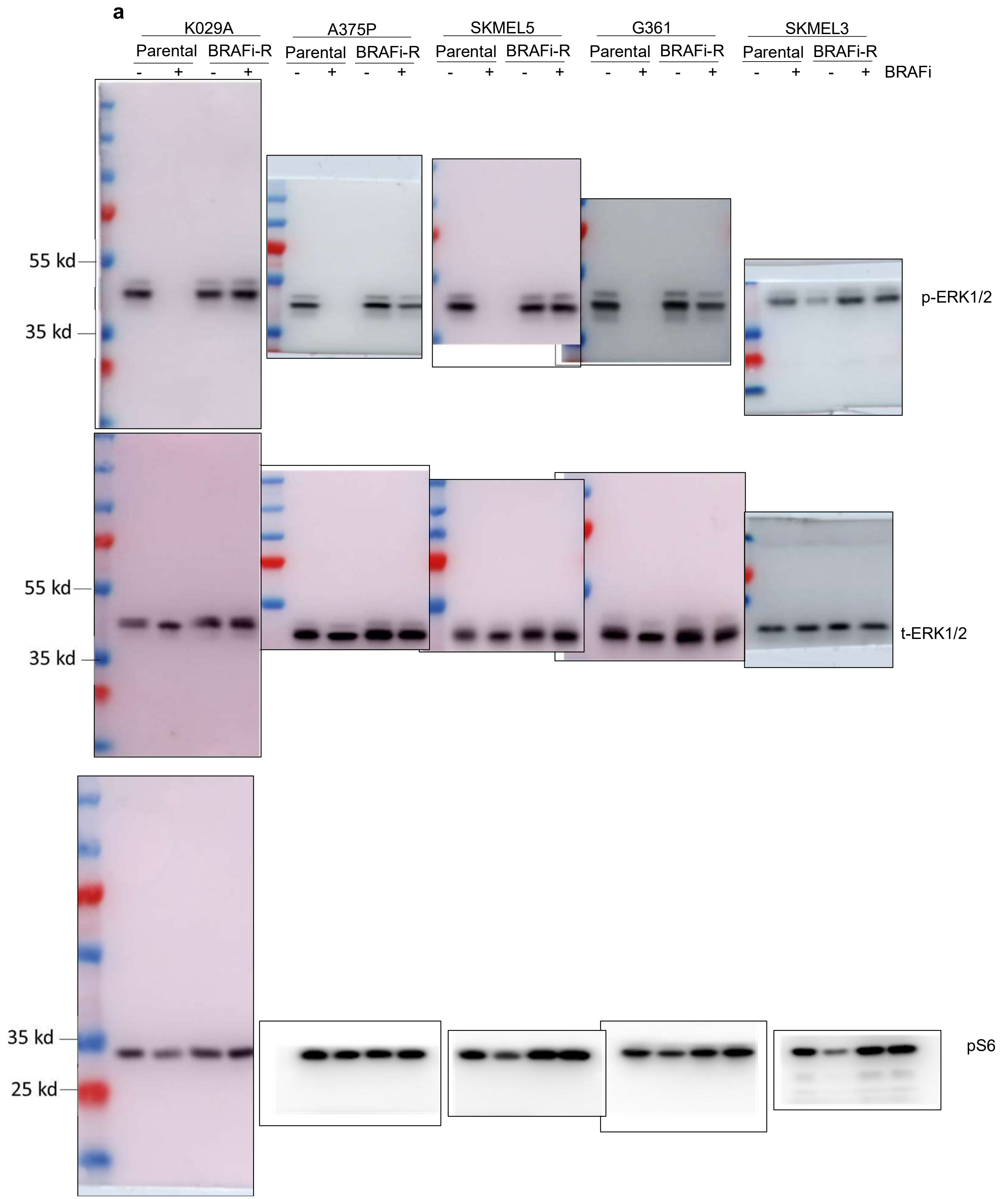

Extended Data Fig. 3

j

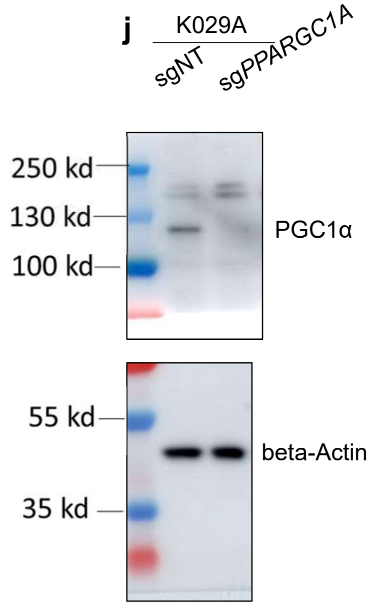

k

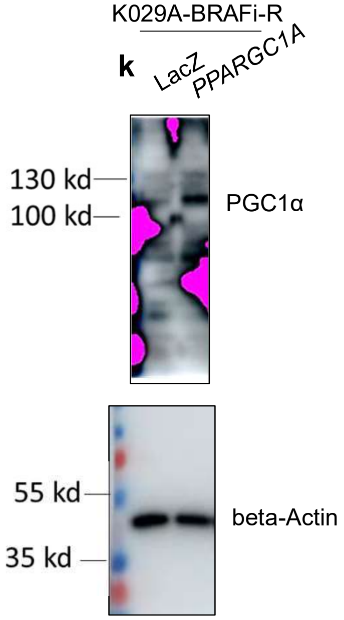

l

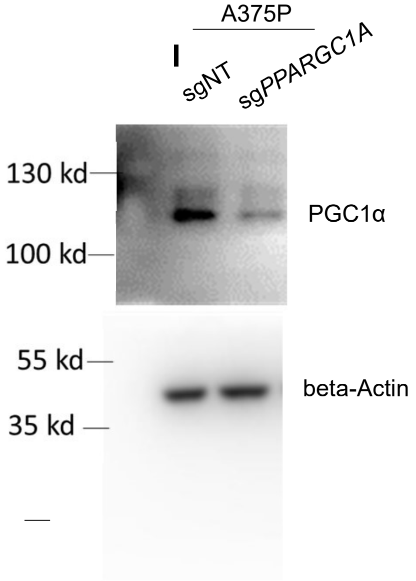

Extended Data Fig. 6

d

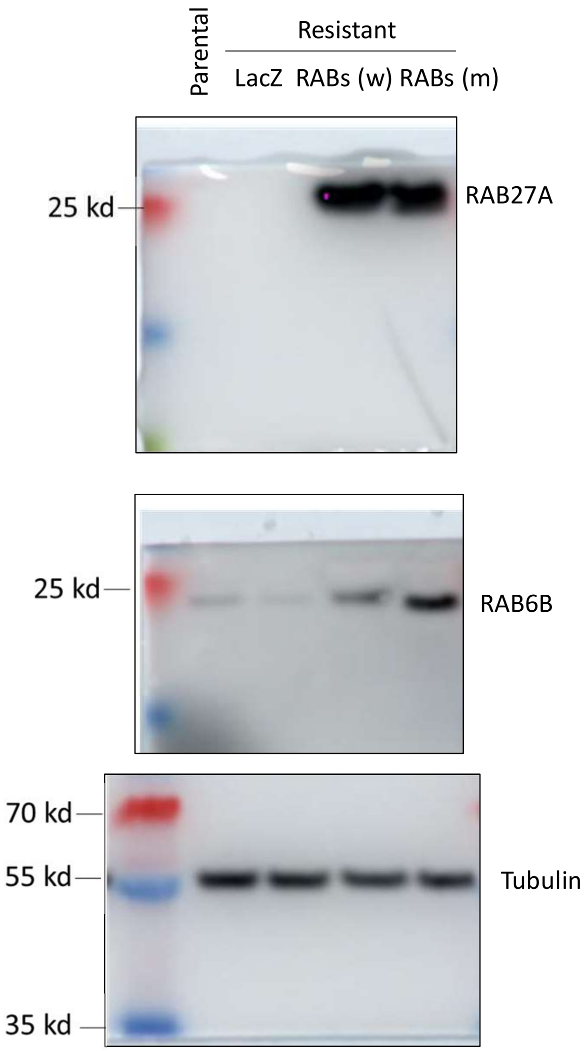

Extended Data Fig. 8

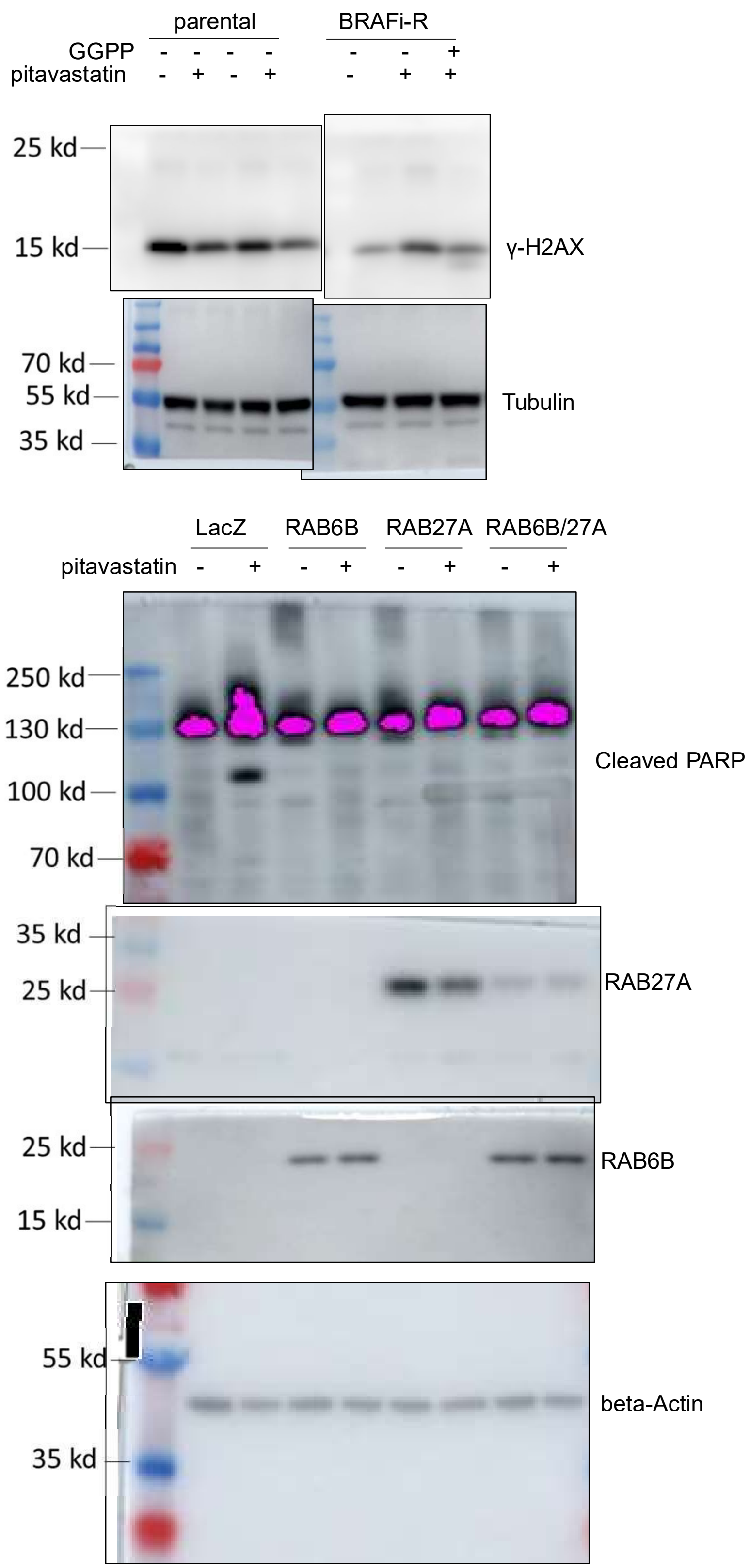

Extended Data Fig. 10

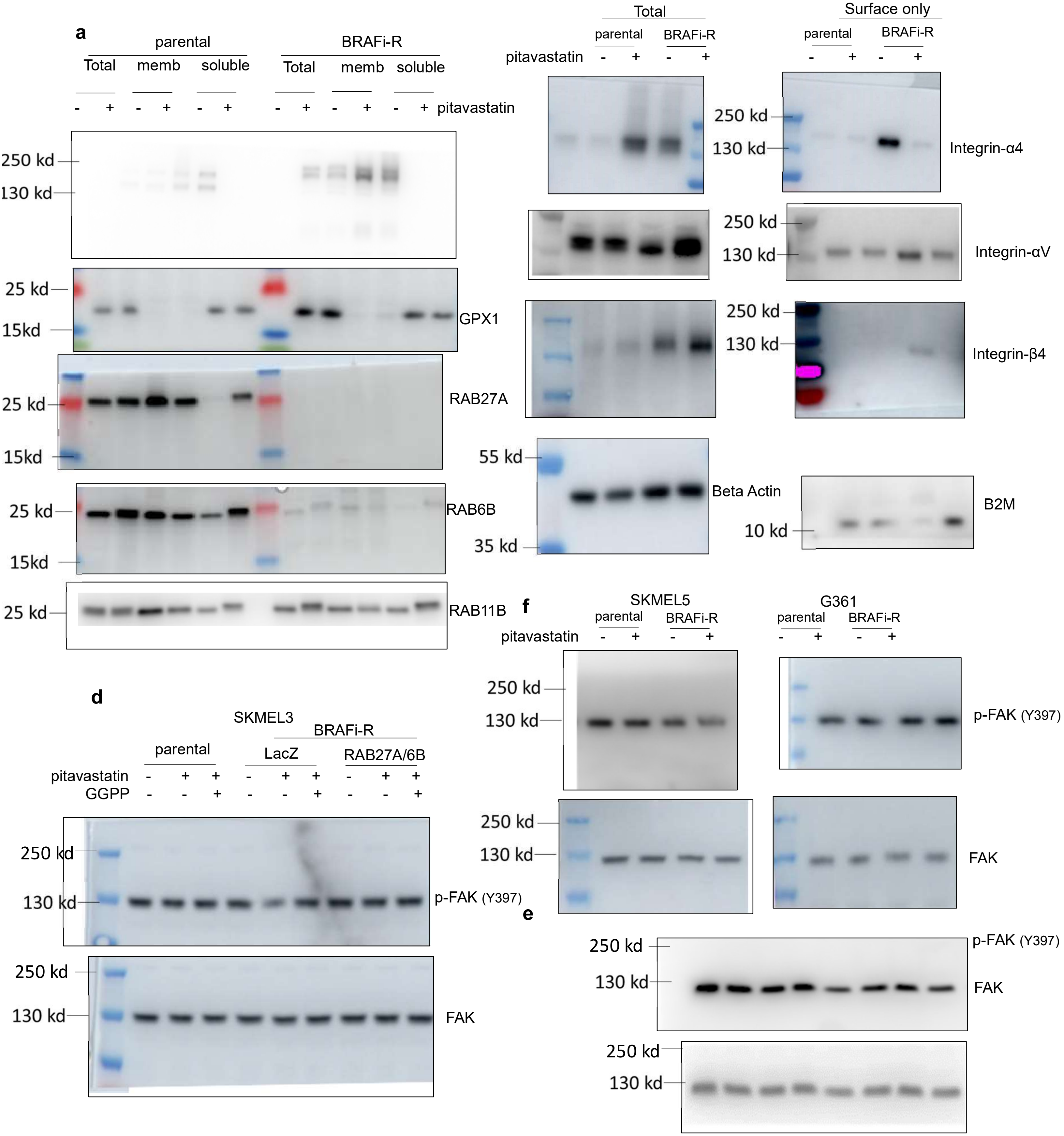

Extended Data Fig. 11

d

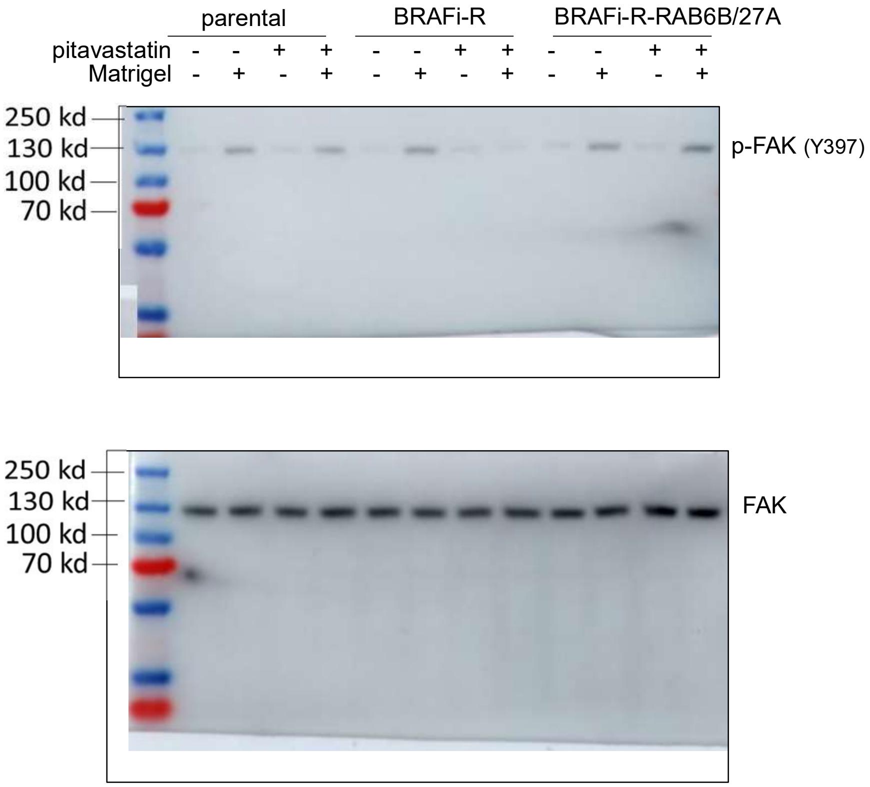

Supplement: Supplementary file 7 — Source Data [file 41467_2023_38968_MOESM7_ESM.zip › raw blots supplemental.pdf]
